# Navigating the Multilayered Organization of Eukaryotic Signaling: A New Trend in Data Integration

**DOI:** 10.1371/journal.pcbi.1003385

**Published:** 2014-02-13

**Authors:** Tapesh Santra, Walter Kolch, Boris N. Kholodenko

**Affiliations:** 1 Systems Biology Ireland, University College Dublin, Belfield, Dublin, Ireland; 2 Conway Institute of Biomolecular and Biomedical Research, University College Dublin, Belfield, Dublin, Ireland; 3 School of Medicine and Medical Science, University College Dublin, Belfield, Dublin, Ireland; National Cancer Institute, United States of America and Tel Aviv University, Israel, United States of America

## Abstract

The ever-increasing capacity of biological molecular data acquisition outpaces our ability to understand the meaningful relationships between molecules in a cell. Multiple databases were developed to store and organize these molecular data. However, emerging fundamental questions about concerted functions of these molecules in hierarchical cellular networks are poorly addressed. Here we review recent advances in the development of publically available databases that help us analyze the signal integration and processing by multilayered networks that specify biological responses in model organisms and human cells

Eukaryotic cells respond to a myriad of external and internal cues via a multilayered signaling network. At the top layer of this network, there are plasma membrane receptors which sense changes in the surrounding environment and play important roles in the communication between cells and tissues. Upon activation, these receptors trigger multiple interweaved signaling pathways which operate via protein-protein interactions (PPI) and posttranslational protein modifications (PTMs), such as phosphorylation and ubiquitination, to generate specific biological responses. Many of these responses include changes in gene transcription, which are controlled throughthe modulation of transcription factor (TF) activities. Activated TFs instigate chromatin remodeling and regulate the production of messenger RNAs (mRNAs), which contain the protein coding regions of the genes. Subsequently, mRNAs are translated into protein molecules. The production, degradation, and translation of mRNAs is delicately regulated by a network of non-coding RNAs, which include micro RNAs (miRNAs) and small inhibitory RNAs (siRNAs). This hierarchical structure is intertwined by a plethora of crosstalks, feedback, and feedforward loops connecting signaling PPI and PTM with transcriptional and translational regulation [1].

## Rapid Growth of Specialized Databases

With recent, rapid advances in modern -omics techniques, our ability to acquire vast amounts of biological data increasingly exceeds our ability to interpret these data. However, the main advances were made in the identification and mapping of the components of signal transduction networks, and these repositories have not translated into understanding how interactions between the components generate network functions and specific outputs. It is still poorly understood how signals are processed and converted into physiological or pathological responses. The prolific output of the -omics technologies has been matched by an ever-increasing number of databases that organize data on biological molecules and their interactions in human cells and in model organisms, such as yeast, *E. coli*, *C. elegans*, *Drosophila*, and others. For example, IntAct, STRING, HPRD, BioGRID, WI8, DroID, YEASTRACT, and SGD [2]–[9] store curated information about protein interactions; PHOSIDA, PhosphoSitePlus, PhosphoELM, NetPhosK, NetworKIN, PREDIKIN, and Scansite [10]–[15] accumulate knowledge about protein phosphorylation and increasingly also about other PTMs; EdgeDB, REDfly, JASPAR, ENCODE, PAZAR, ABS, ORegAnno, and others [16]–[22] provide information about transcriptional regulatory interactions; miRBase, PutMir, Miranda, TargetScan, and miRecords [23]–[27] contain information on miRNAs and mRNA targets of miRNAs; and PutMir, TransmiR, and ENCODE [19], [25], [28] supply information about TFs regulating miRNA expressions. Many of these databases are highly comprehensive in their specialized areas, yet they do not provide an integrated picture of how multiple layers of biological regulation (PPI, PTM, TF-DNA interactions, and transcriptional and translational feedbacks) cooperate to enable the signal integration and processing that determine cellular responses.

To understand the coordinate action of different types of interactions that form multilayered signaling networks, we need to systematically integrate heterogeneous interaction data from the literature and specialized databases. Pioneering efforts have brought us the KEGG [29] and Reactome [30] databases, where signaling and metabolic pathways of several model organisms are reconstructed by curating and integrating PPIs, PTMs, and enzymatic reactions. In Reactome, the reconstituted pathways are peer reviewed by experts in the field, which increases the reliability of the data. The pathways are mapped to other, less studied organisms based on sequence similarities of corresponding components. This approach has revealed how different signaling and metabolic pathways function individually and as an integrated system by communicating with each other. However, the scope of KEGG [29], Reactome [30], and the more recent SPIKE [31] database is limited to signaling and metabolic pathways, ignoring transcriptional and translational regulation. Yet, many components of these pathways control transcriptions and translation, thereby initiating new layers of molecular interactions.

## Capturing the Multilayered Organization of Cellular Networks

Recently developed databases, such as ConsensusPathDB [32], TranscriptomeBrowser [33], InteractomeBrowser [33], [34], and SignaLink2 [35], aim to link signaling pathways to downstream transcriptional regulations by systematically integrating protein-DNA interactions with PPI, PTM, and enzymatic reactions. One of the first such databases, ConsensusPathDB, assembles different interaction types by computationally integrating datasets from 31 databases and by manual curation of interactions from the literature (for further detail see [32] and http://cpdb.molgen.mpg.de/). In addition, ConsensusPathDB contains drug target interactions (collected from pharmacological databases, such as PharmGKB [36], TTD [37]–[40], and Drugbank [41]) to facilitate drug discovery research.

Integrating large volumes of heterogeneous datasets from multiple sources may decrease the overall data quality. Many databases (e.g., PHOSIDA [10], NetPhosK [42], and STRING [2]) store interactions which were predicted by computational means (e.g., by text mining) or from noisy high-throughput datasets. These types of interaction data are prone to errors, and therefore quality control is a crucial factor in data integration. A common approach to quality control is to assign a confidence score to each interaction, which can be used to filter out less reliable interactions. In ConsensusPathDB [32], the confidence score is calculated based on gene ontology and pathway annotations and network topological features. The data retrieved by ConsensusPathDB can be downloaded in standard BioPAX [43] and PSI-MI [44] formats and can also be imported into network analysis and visualization tools, such as Cytoscape [45]. However, ConsensusPathDB does not contain information about posttranscriptional interactions between miRNA and mRNA molecules.

One of the first databases that integrated transcriptional and posttranscriptional (mRNA-microRNA) interactions with other types of biochemical interactions was TranscriptomeBrowser [33], [34]. Although TranscriptomeBrowser was originally designed to identify transcriptional signatures of co-regulated genes from publically available microarray databases, it has a default plugin called InteractomeBrowser [33], [34] that integrates heterogeneous interaction data. Using a gene list as input InteractomeBrowser searches a large number of public databases and the literature sources and retrieves (i) computationally predicted transcriptional interactions, (ii) potential regulatory interactions inferred from ChIP-seq experiments, (iii) literature-curated transcriptional interactions, (iv) predicted posttranscriptional regulation by micro-RNAs, (v) phosphorylation interactions, and (vi) protein binding interactions. Currently, InteractomeBrowser retrieves data from nine different databases and displays it as a network (for further details see http://tagc.univ-mrs.fr/tbrowser/). The layout of the network is designed to group molecules together based on their subcellular localizations. These interactions can be downloaded in different formats, e.g., XML and GINML, for further analysis. The XML format enables the user to import downloaded data into Cytoscape [45], and the GINML format allows the retrieved networks to be imported in the Boolean network simulation platform GINsim [46]. Although, TranscriptomeBrowser [33], [34] encompasses more signaling layers than ConsensusPathDB [32], it uses fewer sources (nine databases) than the latter (31 databases). Additionally, it lacks a systematic quality control measure, which prevents users from filtering out unreliable interaction data. However, the authors of TranscriptomeBrowser pointed out that a new plugin for quality control purposes will be introduced [40].

**Figure 1:**
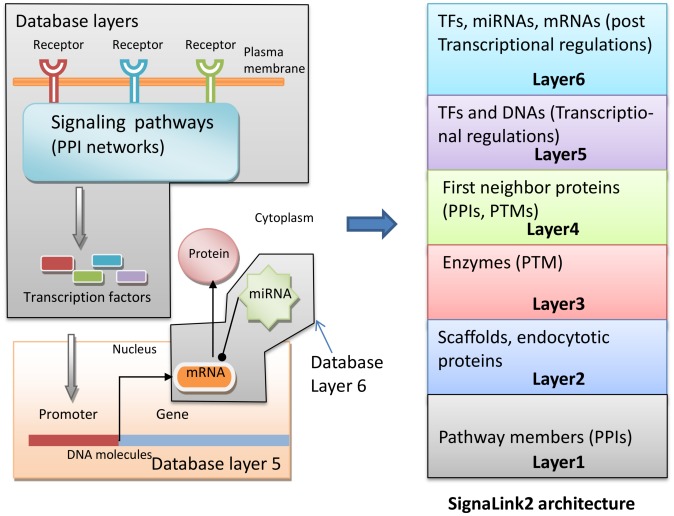
The multilayered architecture of the SignaLink2 database represents the hierarchical organization of signaling pathways.

A recent notable addition to the arsenal of integrated databases is SignaLink2 [35], which systematically integrates PPI, PTM, transcription regulation, and posttranscriptional interactions in one platform. It focuses on seven key signaling pathways, including receptor tyrosine kinase, TGF-ß (transforming growth factor beta), WNT/Wingless, Hedgehog, JAK/STAT, Notch, and NHR (nuclear hormone receptor) pathways. SignaLink2 embarks on the reconstruction of multilayered architectures of these pathways in three different organisms, humans, *D. melanogaster*, and *C. elegans.* For this purpose, it implements a multilayered database architecture (Figure 1) and a promising platform for systematic data integration. The first layer forms the core network based on manually curated PPIs. The second layer contains manually curated interactions involving scaffolds, endocytotic proteins, and the components of the core pathways. The third layer represents interactions that modulate pathway components via PTMs, e.g., kinases, phosphatases, ubiquitin-ligases, and peptidases. Layer four encompasses the directed PPIs where a target protein is in the core pathway(s), while the other protein interacts with it. The directions of these PPIs were inferred based on domain interaction data [47]. The next two layers contain transcriptional interactions between TFs and DNA, and interactions involving miRNAs, such as posttranscriptional miRNA-mRNA regulation and TF-miRNA interactions. Additionally, a large number of undirected PPIs acquired from high-throughput datasets are also provided. The multilayered representation of interaction data allows users to discover inter-pathway crosstalk and feedback mechanisms, which operate via transcriptional, posttranscriptional, and translational mechanisms.

Despite the complex and multilayered architecture of its underlying database, SignaLink2 provides a simple and intuitively clear user interface to search and retrieve information. On the main page (http://signalink.org), it offers a search tool, which allows users to retrieve interactions involving a gene or protein of interest. The retrieved interactions are organized according to their signaling layers and are visualized as a network in the same page. In the download page (http://signalink.org/download), users can retrieve entire pathways and the crosstalk mechanisms between these pathways. To discover multilayered crosstalk between two signaling pathways, the user selects two pathways, an organism, and the signaling layers of interest, and the database retrieves the relevant interactions. Information regarding two additional pathways (NRF2 [48] and the autophagy pathway), which are currently under development, can be accessed from the tools page (http://signalink.org/tools) where two separate user interfaces, customized for these pathways, are provided to facilitate data retrieval. On the same page (http://signalink.org/tools), SignaLink2 also provides two additional tools, PathwayLinker and SignaLog. PathwayLinker retrieves the first neighbor interaction network of the queried proteins and visualizes the pathways that involve the proteins in the retrieved network. SignaLog predicts novel pathway components based on orthologue information.

Information retrieved from the SignaLink2 database can be downloaded in several file formats such as BioPAX, csv (comma-separated values), PSI-MI (tab or xml), Cytoscape, and SBML. The data can also be exported to Boolean pathway simulators, such as CellNetOptimizer [49]. As a measure of data quality, SignaLink2 provides multiple confidence scores for each interaction. For PPIs the confidence score is calculated from semantic similarities of the Gene Ontology (GO) terms, for TF-DNA interactions it is calculated from the position matrix values, for human PPI interactions it provides PRINCESS scores [50], and for all other interactions the original scores from source databases are provided. How to use these scores to control data quality is left to the user. While this provides great flexibility for expert users who can select the most appropriate type of confidence score to filter certain types of interaction data, these choices are likely to pose difficulties to nonexpert users. Therefore, a compound confidence score that summarizes the various confidence measures would be a useful feature.

## Using Heterogeneous Interaction Data in Drug Discovery

One of the main objectives behind integrating heterogeneous interaction data is to understand the mechanistic details of how different pathways modulate each other's activities via PPI, PTM, and transcriptional crosstalk [51]. Such knowledge is crucial for pharmacological research. For instance, when cells are treated with a drug that binds to and inhibits the function of its target protein(s), the effect of the treatment propagates via protein interaction networks into the transcriptional and posttranscriptional interactions. To fully apprehend the effect of a drug, it is necessary to understand the multilayered architecture of biochemical networks. Furthermore, the process of drug discovery and validation is expensive and time consuming. Currently, it focuses on inhibiting a single target with the highest possible efficacy and specificity. Network effects are not considered. The price of this neglect is high, often contributing to drug attrition in later, even more expensive phases of drug development. However, it is experimentally difficult to include network effects in the drug discovery and validation phase. A possible solution is to simulate such experiments computationally, rather than performing them in wet labs. This requires developing computational models of multilayered cellular networks to replicate their response dynamics with reasonable accuracy. Such models will potentially be useful not only for understanding why drugs work, but also why they stop working, and how drug resistance can be overcome.

In addition to the elimination of drugs from cells by export pumps, mechanisms emerging from network design features, such as robustness and adaptation, are now drifting into the limelight. The exact contribution of network-based mechanisms is unknown, but may be substantial given that the network negative feedback and crosstalk motifs, which can cause drug resistance, are common [52], [53]. Computational models of multilayered biochemical networks will provide analysis tools and new insights into how these feedback loops and pathway crosstalk cause drug resistance [54]. Although some databases (SignaLink2 and ConsensusPathDB) discussed in this paper allow users to integrate their data contents into simplistic Boolean simulators, using these data for a more detailed, mechanistic-based modeling approach is not straightforward. Firstly, many databases are limited to a few pathways and layers of signaling mechanisms (see Table 1 for a detailed comparison of the scopes of different databases). Secondly, many of these databases do not annotate different types of interactions in sufficient detail. For instance, SignaLink2 does not differentiate between different types of PTMs, such as phosphorylation, dephosphorylation, ubiquitination, deubiquitination, glycosylation, and cleavage. All PTMs are represented under one category (“post-translational modification”). The knowledge of the “type” of each PTM is necessary for effectively simulating the dynamics of a signaling pathway using ordinary differential equations (ODEs), which allow dynamic simulations of biochemical reactions and a mechanistic analysis of signal transduction pathways. Thirdly, data quality may be a potential concern. Although most integrated databases implement some quality control techniques, the effectiveness of these techniques is yet to be tested. Finally, the topologies of biochemical pathways and the mechanisms by which they communicate with each other are often tissue specific. Currently, databases do not allow users to retrieve tissue-specific interaction networks, thereby potentially limiting the usefulness of the retrieved data for mechanistic modeling.

**Table 1:** Comparison of different databases that integrate heterogeneous interaction data.

Databases	PTM	PPI	Metabolic	TF-DNA	miRNA-mRNA	Drug Target	No. of Species	Scope
Reactome	Yes	Yes	Yes	No	No	No	49	Genomewide
KEGG	Yes	Yes	Yes	No	No	Yes	2,675	Genomewide
SPIKE	Yes	Yes	No	No	No	No	1	28 pathways
CPDB	Yes	Yes	Yes	Yes	No	Yes	3	Genomewide
IBR	Yes	Yes	No	Yes	Yes	No	1	Genomewide
SIGLK2	Yes	Yes	No	Yes	Yes	No	3	7 pathways

CPDB, IBR, and SGLK2 represent ConsensusPathDB, InteractomeBrowser, and Signalink2, respectively. “Yes” indicates that a database includes a certain interaction, and “No” indicates that it does not. Note that the Reactome and KEGG databases contain mostly human and *E. coli* (in the case of KEGG) interaction data and map these interactions in other species based on gene orthology.

## What Next?

The above example of drug discovery is just one of many applications where truly integrated databases could be useful. While there are many more biological and biomedical questions which would greatly benefit, two grand challenges stand out. One is the functional interpretation of genetic and genomic alterations. Next-generation sequencing is now cheap and powerful enough to make the sequencing of human genomes a clinical routine test [55]. Thus, while we are accumulating genetic data at breakneck speed, we are struggling with our limited ability to actually understand what genetic variations and aberrations mean for the patient and how they affect physiological and pathological processes. This means we will need to find new ways to study connections between the relatively static genomic changes and their effects on biochemical and metabolic networks that are dominated by dynamic processes that belie the linear relationships of genetics. The other grand challenge is to understand what we currently call crosstalk between biological pathways. Even in the -omics age the functional modules of biological networks which we call pathways are largely defined from a historical perspective stemming from the time where we worked on one protein at a time (often a lifetime). As a result the pathway concept tends to reflect the history of their discovery more closely than the actual functional connections. However, what we have learned early on is that the interaction between pathways often produces highly nonlinear effects leading to synergistic or antagonistic effects of combinations of drug or growth factors. Understanding such effects obviously could revolutionize both practical applications as well as fundamental biological research. For instance, we could apply this knowledge to the rational design of combination therapies or to gain new insights into interactions between inflammatory cytokines that can escalate to life-threatening conditions.

At the moment we are lacking systematic approaches to each of these grand challenges. Integrated databases will be a cornerstone of developing them. How can we achieve this goal? We will need not only more integration between more things, but primarily we will need more efficient integration. Instead of just linking data we will need to design semantics that, like in a language, instill meaning into a string of linked facts or words. Semantic web tools are finding their way into biology and hold great promise for accomplishing data linking [56], [57]. However, a critical issue is that data linking needs to go hand-in-hand with data filtering to generate useful information. In a language the message is conveyed by the contextual filtering of the possible meanings of the assembled words rather than by the linkage itself. Depending on what we want to find out we apply different filters and different combinations of filters that dynamically change as the conversation evolves. Thus, the ideal database will not only perform semantic linkage, but also dynamic semantic retrieval filtering when queried for different purposes and in different contexts. We basically want the database to give us a human answer to a human question. That is a difficult task comparable to facial recognition, which is routine for humans but really challenging for computers. But that feat is only the beginning. We also need to integrate the databases with analysis tools. There are rudimentary beginnings as discussed above. Ideally, we would like to seamlessly plug data retrieved from an integrated database directly into various analysis machines that calculate enzymatic reactions, reconstruct networks, map sensitive nodes or control points, etc. Thus, we are still far from true integration, but at least we are settings beacons of where to go.

As fully integrated databases have only started to be built, time will show how these databases will change the research and computational modeling landscape. To facilitate computational modeling, integrated databases need to provide dynamic linkage to specialized databases that store quantitative kinetic data on the time course of phosphorylation or other protein modifications for multiple different sites of signaling proteins and enzymes. Then, using semantic and other links between databases, mathematical models can be properly calibrated, and predictive computer simulations would allow us to find the routes and relative intensities of signal flows following a variety of external cues processed by cell surface receptors. This will help us understand cellular responses and phenotypic behavior. A largely understudied problem is the combinatorial complexity of signaling by multi-domain proteins and protein complexes [58]–[60]. Different domains on the same protein can initiate signaling pathways that propagate distinct cellular responses. Owing to the multiplication of different possibilities, interactions between domains, proteins, and protein complexes generate myriads of feasible molecular species, which no database can account for. Yet, integrated databases can tell us whether protein interactions are competing or independent, and how these interactions depend on posttranslational modifications of interacting proteins. As such data are becoming available, integrating this information with interaction data can help us formulate the rules of biochemical interactions. These rules will describe both feasible and improbable classes of interactions to allow rule-based representations and computational modeling of cellular signaling networks. These rule-based models incorporate individual phosphorylation sites on multiple proteins, enabling mechanistic explanation of temporal phosphoproteomic data in the foreseeable future [60]–[63].

## Conclusion

Overall, integrated databases such as ConsensusPathDB, InteractomeBrowser, and SignaLink2 are noteworthy initiatives in reconstructing a global multilayered picture of cellular signaling systems by integrating heterogeneous interaction data from multiple sources. However, integrated databases have a long way to come from their current state, before we can effectively use them to develop a quantitative, mechanistic understanding of multilayered cellular networks at realistic complexity. In particular, integrated databases should include more of already available information. For example, none of the databases named above integrate epigenetic regulations (such as modulation of gene regulation via chromatin remodeling) and mutation data, although this information is increasingly available from such sources as the ENCODE [19] and COSMIC [64] projects. Moreover, integrated databases need to keep up with our requirements to mechanistically understand biochemical networks and their multilayered organization. Although our state of knowledge is incomplete, it is rapidly evolving with the acquisition of new information. It seems appropriate to conclude with a Winston Churchill quote: “Now this is not the end. It is not even the beginning of the end. But it is, perhaps, the end of the beginning.”
